# Current Understanding of the Relationship between Blood Donor Variability and Blood Component Quality

**DOI:** 10.3390/ijms22083943

**Published:** 2021-04-11

**Authors:** Narges Hadjesfandiari, Mona Khorshidfar, Dana V. Devine

**Affiliations:** 1 Canadian Blood Services Centre for Innovation, Vancouver, BC V6T 1Z3, Canada; narges@chem.ubc.ca; 2 Centre for Blood Research, University of British Columbia, Vancouver, BC V6T 1Z3, Canada; mona.khorshidfar@gmail.com

**Keywords:** blood transfusion, blood product quality, blood donor variability, platelet transfusion, red blood cell transfusion

## Abstract

While differences among donors has long challenged meeting quality standards for the production of blood components for transfusion, only recently has the molecular basis for many of these differences become understood. This review article will examine our current understanding of the molecular differences that impact the quality of red blood cells (RBC), platelets, and plasma components. Factors affecting RBC quality include cytoskeletal elements and membrane proteins associated with the oxidative response as well as known enzyme polymorphisms and hemoglobin variants. Donor age and health status may also be important. Platelet quality is impacted by variables that are less well understood, but that include platelet storage sensitive metabolic parameters, responsiveness to agonists accumulating in storage containers and factors affecting the maintenance of pH. An increased understanding of these variables can be used to improve the quality of blood components for transfusion by using donor management algorithms based on a donors individual molecular and genetic profile.

## 1. Introduction

**Figure 1:**
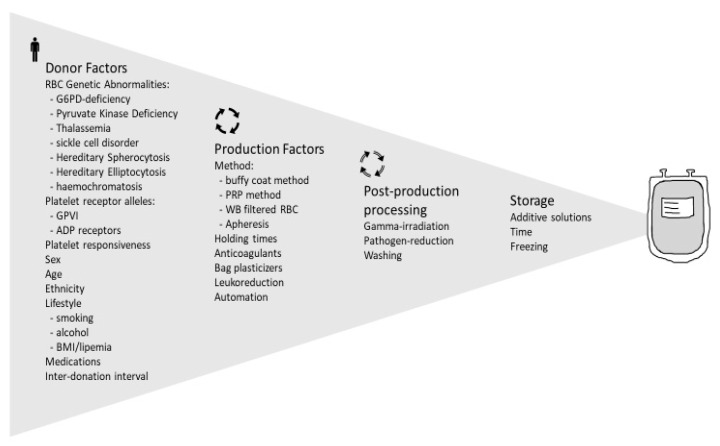
Sources of variability in blood manufacturing. Donor factors contribute substantially to variability affecting blood product quality. RBC (red blood cells), G6PD (glucose-6-phosphate dehydrogenase), GPVI (glycoprotein VI), BMI (body mass index), WB (whole blood). Created by Dr. Katherine Serrano.

Blood transfusion is a lifesaving medical procedure and is the single most common medical procedure carried out in hospitals. According to the World Health Organization, some 118.5 million units of blood are collected worldwide annually. Much of this is further processed into components: packed red blood cells, platelet concentrate, and transfusion plasma. For much of the modern history of blood transfusion, the focus was on blood compatibility from the perspective of blood group antigens such as the ABO type of donor and recipient. More recently, the variability in the blood donor population and its impact on the quality of stored blood products has gained appreciation. It is clearer now that all units of blood products are not the same and the characteristics of the donor are reflected in the biochemistry of an individual unit. This in turn may affect transfusion efficacy and patient outcomes. Increasingly, the molecular basis of these differences has been determined and their impact on component quality better understood. The intent of this review article is to explore the current state of knowledge of these variations in blood donors that impact blood product quality. We will start with red blood cells, one of the best studied of human cell types, then move to platelets, covering the topics shown in Figure 1.

## 2. Red Blood Cells

Mature red blood cells (RBCs), or erythrocytes, are the most abundant cells in the human body and specialize in transporting oxygen. As erythroblasts and reticulocytes emerge from bone marrow to form mature RBCs, they lose their nuclei, mitochondria, and ribosomes to make room for hemoglobin, the oxygen-binding molecule. Each human RBC contains around 270 million hemoglobin molecules, which consist of a globin, a protein with two pairs of identical polypeptide chains (α2β2), and four heme groups (the iron-chelating structure). Each hemoglobin has the capacity to bind four oxygen molecules via ferrous ions (Fe^2+^) in the heme structures.

The high concentrations of iron and oxygen create a perfect environment for the generation of reactive oxygen species (ROS) in RBCs. While lack of mitochondria helps to alleviate this situation, as they are the major source of ROS in cells, the absence of nuclei and ribosomes impairs the ability of RBCs to synthesize new proteins and replace damaged enzymes and structures. Thus, RBCs have evolved various defense mechanisms to protect themselves against oxidant stress [1].

To prepare RBC units for transfusion, donated whole blood is centrifuged, leukocytes are removed below the limit of 5 million per unit, the RBCs are mixed with an additive solution, and stored in a gas-permeable storage bag mainly made of polyvinylchloride (PVC) and di(2-ethylhexyl) phthalate (DHEP) at 4 ± 2 °C until transfusion. RBC shelf life varies from 21 to 49 days depending on the additive solution and jurisdictional regulations [2].

During storage, RBCs undergo biochemical and cytoskeletal changes collectively known as the storage lesion. These changes include, but are not limited to: reduced 2,3-diphosphoglycerate (2,3-DPG) and ATP levels; increased lactate level; changed ion concentrations, for example, increased intracellular Ca^2+^ and extracellular K^+^; reduced concentration and/or activity of CD47; increased exposure of phosphatidylserine; increased concentration of oxidized proteins and lipids; increased damage to proteins, membranes and cytoskeleton; altered phospholipid organization; changed RBC shape; generation of microvesicles; reduced deformability; increased tendency to aggregation and endothelial adhesion; increased susceptibility to phagocytosis and hemolysis; and an accumulation of biomolecules with proinflammatory and immunomodulatory effects in the storage medium [3].

These changes compromise the major task of oxygen delivery and can also lead to adverse effects in recipients such as inflammation, hypertension, vascular injury, renal insufficiency, and organ failure [4]. These events are mainly mediated by cell-free hemoglobin and the subsequent release of hemin (heme with Fe^3+^), which translocates to extracellular spaces, interferes with nitric oxide and oxidant reactions, oxidizes membrane lipids and lipoproteins, activates innate immune responses in macrophages and monocytes, and changes the cell metabolism [4].

Based on the changes that occur during storage, stored RBC units are generally classified into two groups: “young” (<14–21 days) and “old” (>21 days) [5]. The storage lesion impacts the fate of RBCs after transfusion as evidenced by significantly higher post-transfusion recovery (PTR) of young RBCs (stored for 0–10 days) compared to older RBCs (stored for 25–35 days) [6]. Similarly, there are smaller increases in hemoglobin concentration (Hb increment) following the transfusion of long-stored RBCs compared to those stored for a shorter period [7].

Any effect of RBC storage age on clinical outcomes, however, has not been as straightforward [8]. Majority of early retrospective studies did not observe an association between RBC storage age and the investigated clinical outcomes, such as length of stay in hospital/ICU, renal failure, mechanical ventilation, and mortality [9]. An association between the rate of pneumonia incidence and RBC storage age was observed in some studies [9]. In 2008, Koch et al. reported that transfusion of RBC units with a storage age of more than 14 days increased the relative risk of postoperative death by 30% [10]. Despite some drawbacks in the study design, their findings fueled concerns over the efficiency and safety of long-stored RBCs.

Yet, successive clinical trials have not found any correlation between RBC storage age and post-transfusion morbidity/mortality. For example, transfusion of fresh RBCs to premature infants did not result in improved clinical outcomes (evaluated by death and common neonatal morbidities) compared to average blood bank RBCs (ARIPI Trial) [11]. A 4-year randomized clinical trial on a general hospital population also showed that transfusion of fresh blood did not reduce mortality rate (INFORM Trial) [12]. A 10-year retrospective study on ICU patients did not find any association between RBC storage age and mortality, nor length of hospitalization [13]. Further, transfusion of fresh RBC (stored ≤10 days) was not found to be clinically superior to transfusion of older blood (stored ≥21 days) in critically ill adults (ABLE Trial) [14], pediatric patients [15], and patients undergoing cardiac surgery (RECESS Trial) [16]. Similarly, a recent study found that transfusion of old RBCs (stored >14 days) did not increase mortality in adult isolated traumatic brain injury patients [17]. There are limitations and imperfections in the design, experimental models, and consistency of methods/standards in all these observational and prospective studies, complicating their utility in decision making. Further, due to safety and ethical considerations, “old” RBC units used in clinical trials are rarely older than three or four weeks. RBC units with 35–42 days of allowable storage are approved in many jurisdictions and are commonly transfused, particularly in centres with the redistribution programs of blood products from smaller centres to the ones with a high volume of transfusion. Therefore, clinical studies using such products in comparison with fresh RBC units would be relevant and beneficial.

Nonetheless, the findings lead to the conclusion that the quality of stored RBCs and clinical outcome of their transfusion cannot be solely determined by storage time. Not all RBCs age the same, and donor differences play an important role. In this section of our review, some donor factors affecting the quality of RBCs are outlined with an overview of the changes at the molecular level, from enzymatic polymorphism and hemoglobin structure/function variants to cytoskeletal elements and membrane proteins.

The additive solution formulations [18] and the production method [19] of RBC units are known to impact the quality of stored RBCs and are not part of this review.

### 2.1. Genetic Abnormalities Affecting RBC Quality

Some of the more common genetic disorders that affect RBC quality are discussed in this section. There is a relatively high prevalence of heterogenic variants of these disorders; many carriers are asymptomatic or have mild symptoms, and it is not uncommon for them to be unaware of their conditions. These individuals are generally accepted as blood donors by many jurisdictions, as long as they feel well at the time of donation and meet the general donation requirements, in particular the hemoglobin threshold.

#### 2.1.1. Enzyme Polymorphism

##### Glucose-6-Phosphate Dehydrogenase (G6PD) Deficiency

Glucose-6-phosphate dehydrogenase (G6PD) is the rate-limiting enzyme in the pentose phosphate pathway (PPP), which generates pentose and reduced nicotinamide adenine dinucleotide phosphate (NADPH). NADPH is essential for producing reduced glutathione (GSH) from GSSG (oxidized form). GSH plays a major role in protecting RBCs from ROS by acting as the reducing agent in multiple reactions. NADPH is also required for the activity of several antioxidant enzymes, such as catalase, peroxiredoxins, and the thioredoxin reductase system.

Due to an insufficient capacity for NADPH production, G6PD-deficient RBCs are more susceptible to oxidative stress and the resulting hemolysis. G6PD deficiency is the most common human enzyme deficiency, making it relevant to blood donation quality and safety, particularly among the ethnic groups with higher prevalence, such as sub-Saharan Africans (7.5%), Middle Easterners (6.0%), and Asians (4.7%) [20]. A recent study of around 400 randomly selected blood donors in Bangkok, Thailand showed that 7.7% of them were G6PD-deficient [21]. With hundreds of molecular variants, there is a spectrum of clinical presentation. While some patients suffer from chronic hemolysis (Class I), others may not be aware of this deficiency, and others may experience episodes of hemolysis only when exposed to oxidative stress, for example, particular medicines and plants or infections (Class II and III).

Unless specifically tested for, blood centres would not be aware of the donor’s G6PD status. According to the WHO guideline on blood selection, individuals with G6PD deficiency with a history of hemolysis must be deferred permanently. G6PD-deficient donors without a history of hemolysis can be accepted; however, their units should not be used for neonatal exchange transfusion, intrauterine transfusion, and G6PD-deficient recipients [22]. Some centres have specific policies in place, for example, accepting G6PD-deficient individuals for platelet donation but not whole blood donation [23].

Whether the efficacy and safety of transfusion of a G6PD-deficient unit is comparable to a normal unit has not been clear in clinical use. In a systematic review of studies focusing on recipients of G6PD-deicient RBCs, Renzaho et al. showed that many studies could not provide adequate design and methodological quality. They concluded that there was not enough evidence to exclude G6PD-deficient individuals from donating RBCs [24].

Hemolysis due to transfusion of G6PD-deficient units to relatively healthy stable adults was deemed too low to have a clinical impact [25]. However, there is a risk of harm to certain recipients that should be prevented. Case reports of adverse events were reported in premature neonates [26,27], those with concurrent extra oxidant stress, such as some medications [28], and sickle cell disease (SCD) patients [29]. To prevent alloimmunization complications, SCD patients more frequently receive blood from African origin donors (with a higher chance of being R_0_r or R_0_R_0_), who are also more likely to be G6PD deficient [30]. This puts SCD patients at higher risk especially if they are receiving an oxidant medicine and/or have an infection. A recent study showed that SCD patients who underwent chronic transfusion therapy had a significantly higher clearance of normal adult hemoglobin (α_2_β_2_; HbA) when they received G6PD-deficient units versus normal RBCs. No clinical adverse events were observed in any of the recipients. However, sickle hemoglobin (HbS) and reticulocyte count were higher in the recipients of G6PD-deficient blood [29]. This study did not evaluate PTR, which could be used as a direct evaluation of the most recent transfusion (subject of study); HbA clearance reflects clearance of RBC from all transfused units within the prior four months.

In a recent study, Francis et al. showed that G6PD-deficient RBCs had a significantly lower 24-h PTR compared to G6PD-normal RBCs [31]. Notably, three out of 10 G6PD-deficient transfusions led to PTR less than 75%, i.e., they did not meet the US FDA criterion. This study used autologous blood; it is not clear if plasma profile of a G6PD-normal recipient could change the survival of transfused G6PD-deficient RBCs and therefore give a different transfusion outcome. This study investigated the correlation between PTR and metabolites in different pathways and observed a strong correlation, particularly with those metabolites involved in lipid remodeling, energy metabolism, and nucleotide recovery [31]. Their work also provided valuable insight into some in vitro and metabolomic differences between G6PD-normal and G6PD-deficient RBCs, some of which are listed in Table 1.

Despite the defective anti-oxidant system, G6PD-deficient RBCs manage to preserve levels of storage hemolysis and ATP comparable to those of G6PD-normal RBCs. They have even shown improved rheological properties compared to RBCs from G6PD-normal donors [32]. However, this maintenance of energy and morphology is achieved at the expense of the anti-oxidant system, as demonstrated by a significant increase in ROS levels in G6PD-deficient RBCs (and not G6PD-normal RBCs) when they were mixed with fresh plasma to simulate transfusion [32].

G6PD activity in vivo was reported to have no correlation with the storage hemolysis level, however, the levels of some directly relevant metabolites, such as GSH and NADPH, were strongly correlated with hemolysis-related nodes in metabolomics analysis [33], which discourages one from focusing on a single molecule or marker approach when it comes to determining RBC quality. Francis et al. also observed a large variation in PTR levels of G6PD-deficient RBCs and suggested the significance of other factors such as genetic or environmental, independent of G6PD activity, affecting the quality of even this narrow subgroup of RBCs for transfusion [31].

As stated earlier, there is a high variance in clinical presentation (partly absent) of G6PD deficiency, which makes the policy making regarding such blood donations difficult. While there is an agreement that G6PD-deficient donors with a history of hemolysis must be deferred permanently, there is no known risk associated with donations from mild forms of G6PD deficiency that outweighs the benefit of including this group of donations in inventory, except for high-risk recipients.

Screening of the units to be transfused to select populations such as premature neonates can be a solution. In addition, if there is no shortage in blood bank inventory, G6PD-deficient donors could be assigned to donate only for platelet apheresis or special blood group compatibility. Establishing a general program of G6PD screening at the time of donation is an expensive measure (even if limited to male donors or relevant ethnicities) and cannot be justified where there is no evidence of risk to the general population of recipients.

##### Pyruvate Kinase Deficiency

The second most common inherited enzyme defect affecting RBCs is pyruvate kinase deficiency (PKD). It is relatively rare and estimated (from gene frequency) to affect 1 in 20,000 individuals [34]. The clinical diagnosis rate is significantly lower due to its difficult diagnostic evaluation and heterogenic clinical presentation [35]. Pyruvate kinase catalyzes the last step of glycolysis, which yields one molecule of pyruvate and one molecule of ATP. Lacking mitochondria, RBCs are highly dependent on ATP production by glycolysis. The deficiency in pyruvate kinase and the resulting ATP shortage lead to hemolysis, usually referred to as chronic nonspherocytic hemolytic anemia.

PKD patients are usually too anemic to donate, and they suffer from associated complications; therefore, they are deferred from donation permanently. Asymptomatic and mild PKD heterogenic variants mostly remain undiagnosed, and people with these variants can donate blood as long as they meet the hemoglobin threshold for donation and feel well. Whether RBCs from this group of donors store poorly and/or have any unwanted clinical outcome in recipients has not been reported. This could be due to rarity, the benign character of variants, or donors being misdiagnosed with other types of anemia.

#### 2.1.2. Hemoglobin Abnormalities

Genetic hemoglobin abnormalities can be classified into two general groups [36]: quantitative defects in globin chain synthesis and accumulation or thalassemia syndromes; and qualitative defects in globin structure, including sickle cell disorders, unstable hemoglobin variants, hemoglobin with altered oxygen affinity, and methemoglobinemia (Fe^3+^-containing hemoglobin with, thus, lower capacity for oxygen delivery).

##### Thalassemia

Absent or decreased production of α-globin and β-globin subunits are known as α- and β-thalassemia, respectively. In α-thalassemia, one or more of the four genes of α-globin subunit are absent. One deletion has no clinical significance and is known as a “silent carrier”. Deletion of two genes occurs in “carriers”; this is more common in Asian and African/Black ethnicities, and causes mild hypochromic, microcytic anemia. When three genes of α-globin are deleted, the β chain forms the HbH tetramer. This tetramer is soluble but relatively unstable and precipitates over time. HbH disease is clinically presented as hemolytic anemia but usually does not require treatment. Newborns with all four genes deleted, or α-thalassemia major (Hydrops fetalis with Hb Bart’s), do not survive or in rare cases of survival, depend on lifelong transfusion [36].

Differently, β-thalassemia syndromes are mainly caused by point mutations, with more than 300 alleles described to date [37]. Despite variations, the clinical phenotypes can be classified into two groups: β-thalassemia minor (β-thalassemia trait or heterozygotes), which causes mild to moderate hypochromic microcytic anemia; and β-thalassemia major (homozygotes or compound heterozygotes), which presents as hemolytic anemia with a need for blood transfusion. In contrast to α-thalassemia, where excess β chains could form HbH, excess α subunits in β-thalassemia quickly precipitate and damage RBCs. As a result, β-thalassemia has a more severe presentation.

Thalassemia is more prevalent in Africa, Middle East, India, and regions with endemic falciparum malaria, most likely because of its protective effect against malaria [37] A mild form of α-thalassemia affects up to 20% and 40% of Africans and Middle Easterners, respectively [38]. A study involving 400 Thai blood donors reported that 21% of this population carried thalassemia traits [21]. Thalassemia cases are growing rapidly in North America both in number and diversity due to immigration [38]. A prospective study in Ontario, Canada showed that 54% of individuals (*n* = 664) with microcytic anemia had a form of thalassemia [39]. Some studies in countries with a high prevalence of thalassemia have proposed screening of blood donors for thalassemia traits to ensure provision of blood products that have a high functionality, particularly for susceptible recipients such as children. In a small study in Malaysia, 13 out of 80 blood donors had a type of minor β-thalassemia [40]. Thalassemia screening of blood donors could have another benefit—informing donors of their trait status could help prevent the birth of babies with severe hemoglobinopathies [40,41,42]. Microcytic RBCs have been named as a production challenge for platelet and plasma production as well [40].

Nonetheless, currently, individuals with minor types of thalassemia, including α-thalassemia carriers, silent carriers, and individuals with β-thalassemia minor, are eligible to donate blood in Canada, UK, and many East and South Asian countries if they meet the hemoglobin requirement on the day of donation and generally feel well.

##### Sickle Cell Disorders

An amino acid substitution (hydrophobic valine in place of hydrophilic glutamine) at the sixth residue of the β-globin subunit results in a defective Hb structure known as HbS. People who have the heterozygous variant (HbAS), or sickle cell trait, typically have 40% HbS and 56% to 58% HbA and in general have no symptoms unless they undergo hypoxic conditions [36]. On the other hand, in HbSS or sickle cell anemia, deoxy-HbS polymerizes, forms long rigid fibers that alter the RBC shape from biconcave disc to elongated crescent (sickled). Sickling is reversible by oxygenation; however, repeats of this cycle damage the membrane and shorten the RBC life span, accompanied by hemolysis-mediated endothelial dysfunction and activation of innate immune pathways [43]. As a result of impaired biorheology, higher viscosity of blood, and increased adhesion-mediated vaso-occlusion, sickled cells flow less efficiently through capillaries, leading to episodes of tissue hypoxia and even necrosis and organ damage in HbSS patients [43].

Similar to thalassemia, sickle cell disorders are more common in regions with endemic malaria, in particular Africa and Middle East evolving because of the advantage they demonstrate in protecting RBCs against malaria infection. Approximately 8% of African Americans carry the HbAS variant [44].

People with sickle cell trait are eligible to donate blood in many jurisdictions; however, they are asked to inform the donation centre if they are a carrier. It has been estimated that around 1% of blood donations in the US are unknowingly from donors with the sickle cell trait (HbAS) [45].

Filtration (leukoreduction) of HbAS RBCs can be challenging; RBC production might fail to proceed or lead to a high residual white blood cell count. Some HbAS RBCs are leukoreduced successfully and enter the blood bank [45,46]. Different jurisdictions have different policies in place for the use of HbAS blood. For example, it may be excluded from intra-uterine and neonatal use, or from transfusion to SCD recipients. As discussed above, SCD patients are more likely to receive blood from donors of African descent. This means a higher chance of receiving RBCs from a donor with HbAS blood. Such donations would be less effective and may be harmful as the purpose of blood transfusion to sickle cell patients is almost always reducing the HbS percentage. Point-of-care methods for screening blood for sickle cell trait are available and have dramatically improved compared to classic hemoglobin solubility tests [47].; they are successfully used by some centres before RBC transfusion to vulnerable recipients if it is not an emergency situation [45,47]. That being said, the necessity of such screenings seems to depend on the prevalence of the trait in population. In 2017, a large Canadian hospital stopped a 7.7-year audit of screening RBC units for HbAS before transfusion to HbSS patients. They found that the probability of an RBC unit from a donor with HbAS being transfused to an HbSS patient was too low to justify the cost of screening [48].

There is a paucity of research on the efficacy and safety of HbAS RBCs transfusion [49]. This might be due to the socioeconomical conditions of the regions with higher incidence that cannot afford to invest in such projects. Further, some blood banks in these regions would suffer from blood shortages if they exclude this donor population. Immigration and interethnic marriages will gradually make the studies on HbAS relevant to more regions across the world.

A relatively recent study demonstrated that during cold storage, the percentage of hemolysis and osmotic fragility of HbAS RBCs were significantly higher compared to HbA RBCs when tested after four weeks of storage [50]. No difference was found between the storage hemolysis levels of the two groups at a shorter storage time. Post-transfusion survival of HbAS human RBCs in a mouse model was also consistently lower than that of HbA RBCs only when cells were stored close to expiry. This raised the question of whether the allowable shelf life of RBCs from sickle cell trait donors should be lower than that of RBCs from non-HbAS donors [50].

There are other mutations that affect solubility, stability, and/or oxygen affinity of hemoglobin, as well as posttranslational modifications to hemoglobin structure, which are relatively less common. In Canada—and this is likely to be generally representative elsewhere—if the affected individual feels well on the donation day, meets the hemoglobin requirement, and has no history of associated complications, they are eligible to donate.

Canada, UK, US, and many other jurisdictions currently accept whole blood donations from donors with sickle cell and thalassemia traits but ask donors to inform the centres if they are aware of the condition. Many donors would not be aware of their genetic traits, however. As showcased by the Canadian study for sickle cell trait individuals [48], donor screening might not be currently justified financially, however, with an increase in the sickle cell and thalassemia traits populations in future, the outlook might change. Limitation of screening to the populations with relevant ethnic origins as well as those who have a donation history of a unit that failed leukoreduction by filtration (sickle cell disease) can be a reasonable approach until screening of all donors becomes possible. Similarly, all units aimed for high-risk recipients could be screened for the traits.

As the maintenance of inventory is a constant challenge for many blood centres, exclusion of thalassemia and sickle cell traits cannot be a practical solution. Alternate approaches, for example, the introduction of shorter shelf life for these RBC units, is an avenue to explore.

#### 2.1.3. Genetic RBC Membrane Disorders

##### Hereditary Spherocytosis

Hereditary spherocytosis is the most common inherited RBC membrane disorder. Although there are minor forms that can remain undiagnosed, its prevalence is around one in 2000 to 3000 individuals [51]. Hereditary spherocytosis has been reported all around the world without any specific pattern. Mutations in genes of RBC membrane proteins, including ankyrin, band 3, protein 4.2, α- or β-spectrin, and RhAG, result in some defects in vertical connections between the phospholipid bilayer and membrane skeleton, which causes instability of the membrane and vesiculation of the lipid bilayer and therefore cell sphericity [51]. Less deformation of spheric cells leads to mild to moderate hemolysis in patients and splenomegaly. Individuals with known hereditary spherocytosis are permanently deferred from whole blood donation in Canada, US, and UK, likely to be generally representative elsewhere. Similar to other minor variants, donors with mild symptoms knowingly or unknowingly can donate as long as they meet general donation requirements.

##### Hereditary Elliptocytosis

Hereditary elliptocytosis is another RBC membrane disorder that affects 3 to 5 people in 10,000. It is relatively more common in malaria-endemic regions. RBCs with hereditary elliptocytosis have defective horizontal protein connections of the membrane skeleton, including the spectrin dimer–dimer interaction and the spectrin–actin–protein 4.1R junctional complex [51]. The clinical presentation varies from asymptomatic to mild to severe hemolysis. About 75% of individuals affected by hereditary elliptocytosis are asymptomatic and might not be aware of their condition. This population is deemed to be safe and permitted to donate. Velarcetii et al. reported a case of a frequent donor whose RBCs were investigated on her seventh donation and found to be 80% elliptocytes [52]. There was no hemolysis or hematological disorder detected in the donated unit. In addition, the medical records did not show any adverse events in the previous recipients of her blood. However, there were no data collected on the efficacy of transfusion [52]. An earlier study showed that hereditary elliptocytosis RBCs had a shorter half-life in recipients, and suggested that even in the absence of hemolysis, using hereditary elliptocytosis RBCs for transfusion purposes should be investigated [53].

Hereditary elliptocytosis patients with a history of hemolysis and/or splenectomy are not eligible for donation in Canada and UK, likely to be generally representative elsewhere. Asymptomatic individuals are eligible to donate but are recommended to inform the donation centre.

##### Familial Pseudohyperkalemia

Familial pseudohyperkalemia is a hereditary stomatocytosis disorder where a temperature-dependent abnormality in the movement of ions across the membrane leads to an excessive potassium leakage from RBCs, only apparent at temperatures below 37 °C [54]. Individuals with familial pseudohyperkalemia mostly remain asymptomatic and undiagnosed so they can become blood donors. Cold storage of RBCs from these donors can lead to an accumulation of potassium in the units and endanger lives of at-risk recipients, particularly neonates and infants if they receive high volumes of such blood. Strikingly it has been reported that familial pseudohyperkalemia mutation may affect 1:500 European blood donors [54], however, the rarity of case reports suggest that these RBC units must be relatively safe for the general recipient population. The usage of only fresh units (<5 days of storage) and washing RBCs do not seem to result in a safe concentration of potassium in the cold-stored familial pseudohyperkalemia units, therefore, testing RBC units for supernatant potassium levels before transfusion to neonates and infants could be a better approach [54].

#### 2.1.4. Hereditary Haemochromatosis

Haemochromatosis is characterized by iron overload due to an impaired iron regulatory hormone, hepcidin, and the resulting excessive iron absorption from diet. It is a relatively common genetic condition, particularly in people of Northern European descent, with a prevalence of 1 in 300 people [55]. Phlebotomy helps people with haemochromatosis reduce their high levels of serum iron; policies regarding blood donation from this subgroup vary among different blood centres. In Canada, for example, individuals with haemochromatosis can donate blood at the usual interval for whole blood donation, which is every 56 days for males and every 84 days for females. The donors are indefinitely deferred, though, if they have complications, such as liver cirrhosis or heart failure. If a physician assesses the individual’s serum ferritin level to be too high to require more phlebotomy, they can have an outpatient phlebotomy in between donations as long as there is at least a one-week gap between the phlebotomy and blood donation. US blood centres have different policies, but the American Red Cross, for example, currently accepts blood donations from this group. UK blood centres ask donors to contact the centre for further evaluations to determine eligibility. A survey among blood centres in 2013 summarized that approximately one third (11/35) of the participating blood centres (located within 10 European countries and Hong Kong) did not accept donations from haemochromatosis patients and carriers [56].

A comparison of RBCs donated by people with and without hereditary haemochromatosis showed no remarkable differences in the levels of hemolysis, biochemical markers such as 2,3-DPG, ATP and glucose, and soluble immune modulatory factors throughout the 42-day storage. However, haemochromatosis RBCs showed slightly higher levels of phosphatidylserine [57]. A recent study showed that RBC deformability in donors with haemochromatosis, who had no complications and received no treatment, was significantly lower than that of normal RBCs [58]. Shear stress, which is relevant to blood transfusion, impaired RBC deformability in haemochromatosis donors to a higher extent than in normal RBCs [58]. This study suggested that RBCs from asymptomatic haemochromatosis patients might have suboptimal therapeutic effect [58]. Due to toxicity of iron, a safe approach has been suggested to avoid the transfusion of haemochromatosis RBCs to at-risk groups [57]. Such policies are not known to currently exist in the blood centres that accept donors with hereditary haemochromatosis. However, as this group of donors usually become regular donors, identifying and tagging their units seem to be a reasonable approach to ensure providing the optimum quality units for at-risk recipients such as patients undergoing extracorporeal circulation.

All the traits discussed above affect the biochemical and/or biophysical properties of RBC to some extent. When conducting RBC research or clinical assessments, it is important to remember that many trait carriers are unaware of their conditions and are categorized as ‘normal’ blood donors. It is worth mentioning that jurisdictions increasingly include some genetic blood disorders, namely sickle cell traits as part of newborn screening [59]. Although it is possible that not all hospitals disclose the trait status to the newborn’s parents [60], there is a likely trend toward an increased awareness of future donors about their hemoglobin or enzyme variants; blood centres should have clearer policies on blood use from these donors and/or tagging the units.

**Figure 2:**
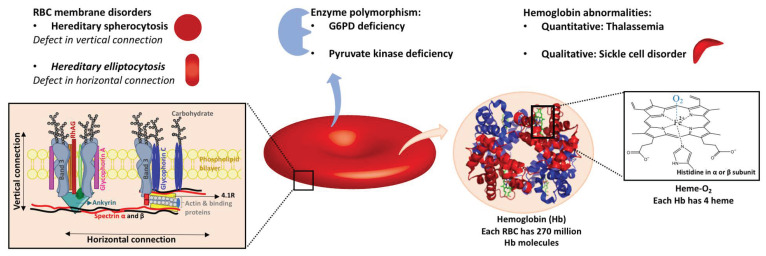
Common genetic abnormalities affecting RBC quality include hemoglobin abnormalities, enzyme defects and RBC membrane disorders. The RBC membrane structure is simplified for clarity. The hemoglobin 3D structure was obtained from the Protein Data Bank-free access.

Figure 2 summarizes the genetic factors affecting red blood cell function that may have an impact on transfusion outcome.

### 2.2. Demographic Factors Affecting RBC Quality

From the complexity of events during the storage lesion, one could imagine that prediction of RBC quality and the transfusion outcomes based on one or two factors cannot be conclusive, as shown by some clinical studies that will be briefly outlined in this section. Identification of the determining factors of RBCs quality, their mechanisms of action and the interplay among them can lead scientists to develop new additives, storage materials or technologies (anaerobic storage [61] is a recent example) toward achieving a high-quality product, and also provides blood centres with some criteria for a better management of donations and possibly more informed processing of whole blood units into a specific blood component.

Much of the data described in this section is from the third phase of the Recipient Epidemiology Donor Evaluation Study (REDS-III). REDS started in 1989 with a goal to “evaluate and improve the safety and availability of the blood supply and the safety and effectiveness of transfusion therapies [62].” REDS-III, as we will see, included research on all elements of transfusion chain from blood donors to recipients. Currently REDS-IV-P with a focus on transfusion to newborns, children, and pregnant women is underway.

#### 2.2.1. Sex

Storage hemolysis has been shown to be higher in male RBCs compared to female RBCs [63,64]. As part of the REDS-III program, Kanias et al. showed that average storage hemolysis was significantly higher in males (0.41 ± 0.29%) than in females (0.35 ± 0.32%) measured at days 39–42 post-collection (*n* = 10,552). Male sex was also associated with higher osmotic and oxidative hemolysis levels [64].

In another study the difference in hemolysis levels of male versus female RBCs was reported to be the largest in the reproductive ages of 30–59 [65]. Further, RBCs from postmenopausal female donors and male donors showed higher mechanical fragility compared to RBCs from pre-menopausal donors, which suggested the significance of sex hormones [66]. In line with these findings, an earlier study has reported an improved resistance to osmotic fragility as well as higher ATP levels in the RBCs with added progesterone in their storage solution [67].

In a recent research in mice models, however, ovariectomy did not have a significant effect on RBC response to osmotic or oxidative stress. Orchiectomy improved PTR and protected cells against hemolysis, and testosterone therapy led to higher levels of osmotic and oxidative hemolysis [65]. The significance of the effect of sex hormones on RBC quality in humans can be different from mice, and more studies are required to clarify this.

Male hemoglobin has a higher affinity toward oxygen than female hemoglobin [68]. In a study of 60 healthy adults, Fähling et al. showed that there is a broad diversity in hemoglobin-oxygen affinity among humans, which is mainly attributed to sex [69]. Levels of testosterone or estrogen have not shown any correlation with the oxygen affinity of hemoglobin, however [70,71]. More studies must emerge to confirm this.

As stated above, RBC quality is affected by much more than one factor. The high analytical power of metabolomics in understanding interwoven pathways can shed light on the mechanisms of sex effect and the disconnections between in vitro and in vivo observations. In a recent metabolomic analysis, a correlation between PTR and epiandrosterone, a steroid hormone that is known to affect glucose metabolism and PPP activity in vitro, has been established [31].

Whether clinical transfusion outcomes are affected by the sex of the donor and/or donor-recipient sex-(mis)match has been the topic of some large retrospective studies, and in brief, one cannot make a strong statement or recommendation based on them [72,73]. RBC transfusions from a longitudinal cohort of 30,503 RBC recipients in Canada showed a statistically significant association between the RBC transfusion from female donors and increased mortality [74]. Shortly after, a cohort consisting of 31,118 patients in the Netherlands reported that transfusion of RBCs from an ever-pregnant female donor led to a higher rate of mortality among male recipients but not among female recipients. On the other hand, transfusions from female donors who were never pregnant were not associated with increased mortality among male or female recipients [75].

On the contrary, data from three cohorts, Kaiser Permanente Northern California (KPNC, 34,662 recipients), REDS-III (93,724 recipients) and Scandinavian Donations and Transfusions (SCANDAT, 918,996 recipients) did not show any increase in mortality following RBC transfusion from female, previously pregnant, or sex-mismatched donors [76]. Differences in the blood banks practices, methodology, statistics models and assumptions are some of the possible reasons of the discrepancies in the outcomes of these studies.

Females, particularly those with a history of pregnancy or receiving blood transfusion or transplants have higher levels of anti-human leukocyte antigen (HLA) or anti-human neutrophil (HNA) antibodies. Transfusion-related acute lung injury (TRALI) occurs during transfusion or shortly (within 6 h) after transfusion of the blood products that contain anti-HLA or anti-HNA antibodies in their (residual) plasma, to recipients with primed neutrophils bearing the cognate antigen. Although the amount of plasma in RBC units is very limited, this product is associated with the largest number of reported cases of TRALI in Canada [77] (Plasma units in Canadian Blood Services are produced only from male donations while sex is not considered in RBC production). It is not clear from the currently available data if RBC transfusion from female donors increase the risk of TRALI in recipients [72]. Most clinical studies involved patients who had received some plasma-containing blood products along with the RBCs, which made the analysis difficult.

#### 2.2.2. Age

Storage hemolysis in female RBCs has been shown to gradually increase with donor age [64,78]. The pattern was found to be different in male RBCs where the storage hemolysis gradually increased with age between the ages of 18 and 45, then decreased in older donors [64]. The magnitude of changes in hemolysis due to the age of donor (limited to 0.04%) was not as large as the effect of sex. The change in osmotic hemolysis due to a donor’s age had a similar pattern to that of storage hemolysis in both sexes while oxidative hemolysis decreased with donor age in both male and female RBCs [64]. Erythropoiesis is reduced with age; RBC count, hemoglobin level, and hematocrit have been found to significantly decrease with age [79]. RBC fluidity and deformability also alters in advanced age as a result of enhanced oxidative stress [80]. The RBC membrane from centenarians had and increased cholesterol/phospholipid ratio and increased integral protein band 4.2 and actin [79].

A retrospective cohort study of 93,726 patients in the REDS-III program showed that hospital length of stay was lower for the recipients of RBC units from young donors (<20 years old) [81]. Analysis of data separately for female and male RBCs gave a similar result. The effect was deemed small and appeared when four or more units of RBCs were transfused [81]. Inflammatory markers change with age, and this can be investigated further in relation to the apparently favorable outcomes from the transfusion of young donor’s RBCs [81]. Interestingly, a cohort study of 30,503 RBC recipients in Canada had observed the opposite effect, namely that RBC transfusion from younger donors was associated with increased mortality [74]. Consistent with these different results, a systematic review of clinical studies could not establish an association between donor age and RBC transfusion outcome [72].

In many jurisdictions, there is no upper age limit for blood donation as long as the donor meets general eligibility requirements. Many blood centres have difficulties in recruiting younger donors and excluding seniors can result in short supply. The minimum age for blood donation eligibility is generally between 16 and 18 years old; low body mass index (BMI) causes a temporary deferral of some young donors.

#### 2.2.3. Ethnicity

As discussed above, some ethnicities have higher prevalence of hemoglobin variants and/or enzyme polymorphism that impact the biochemical and physical properties of RBCs. Recording a donor’s ethnicity is not generally among blood centres policies but the current increasing trend of optional disclosure of ethnicity by donors will provide more data on the significance of ethnicity in relation to the quality of RBCs and blood products, in general. In the REDS-III study by Kanias et al., African American, Asian and Hispanic donors showed higher levels of both storage and oxidative hemolysis compared to RBCs from white donors [64]. However, osmotic hemolysis was in these groups significantly lower. In particular African American donors had remarkable resistance to osmotic fragility, which can be explained by the high prevalence of hemoglobin variants, in particular sickle cell disease and thalassemia traits in this ethnic group.

#### 2.2.4. Lifestyle

In general, characteristics of an unhealthy lifestyle such as physical inactivity, tobacco use, alcoholism, having an antioxidant-poor diet or fatty diet are in line with decreased antioxidant activity and an increased oxidative stress in human cells, including RBCs. To the best of our knowledge, a cross-sectional study of 760 Dutch donors as part of the Donor Insight program is the only large scale analysis of any effect of lifestyle on hemolysis [82]. This study did not find any association between hemolysis level and life style behaviours including moderate-to-intense physical activity, intake of unsaturated fatty acids-rich foods, or intake of saturated fatty acid/cholesterol-rich foods [82]. They observed, however, an association between lipemia and hemolysis level; this factor will be discussed further.

Physical activity has been speculated to have a positive effect on the peroxiredoxin system [83]. Peroxiredoxin forms part of the RBC antioxidant capacity, and its reduced level has been reported in donors with repeated high storage hemolysis [84]. While regular moderate exercise is beneficial for oxidative stress and health, acute intense exercise causes excessive oxidative stress [85].

Lifestyle behaviours apart from those associated with increased risk of blood transmissible pathogens are not part of donor screening or data collection. Some of the more widely investigated life behaviours that affect RBC quality are discussed here.

##### Smoking

Smoking has been associated with increased oxidative hemolysis and decreased osmotic fragility of RBCs [86]. Storage hemolysis levels of RBCs from smokers has been reported to be comparable to that of non-smokers’ RBCs [87].

Carboxyhemoglobin (COHb) levels are significantly higher in smokers [87]. A study in Brazilian blood donors suggested that being abstinent for at least 12 h prior to blood donation decreases the level of COHb, and could be recommended to donors by blood centres. In this population, 6% of donations were from smokers. This study did not observe any differences in hemolysis % and hematological parameters of smokers and non-smokers [87]. Another study also reported no increase in hemolysis level as a result of smoking but observed increased levels of hemoglobin, mean cell volume, and mean cell hemoglobin in donors who smoked 10 to 20 cigarettes per day for at least 3 years [88]. A recent pilot investigation on 100 donations in REDS-III program showed that 13% of units were cotinine-positive, comparable to an estimated smoking rate of 15.5% in the US population [89]. Cotinine is one of the main metabolites of nicotine and widely used as a measure of active smoking. Cotinine-positive RBCs had higher levels of COHb and their transfusion led to lower hematocrit and hemoglobin increment in recipients compared to cotinine-negative RBCs [89]. The REDS-III retrospective cohort study showed no association between hospital mortality or posttransfusion length of stay of recipients and smoking status of RBC donor [81].

Cigarette smoking is associated with increased oxidation in RBCs and as a result high basal activation of anti-oxidant system. In addition, as part of the REDS-III program, Stefanoni et al. showed that cotinine-positive RBCs had increased levels of oxidant stress markers such as PPP metabolites and pyruvate-to-lactate ratio [86]. Increased consumption of dietary antioxidants such as ascorbate, glutathionylation of oxidized sugars and lipid aldehydes, fatty acid desaturation, lipid oxidation, and methionine consumption in the repair pathway were consistent with cotinine-positive RBCs being under oxidative stress [86]. It has been reported that RBCs membranes from smoker donors do not retain normal band 3 and stomatin levels. Secretory apolipoprotein J/clusterin (sCLU), a chaperon protein involved in oxidative injury pathways, was also removed from RBC membranes of smoker donors [90].

Smokers have higher levels of lead and cadmium in their blood, which are mainly stored in RBCs. Concerns over transfusion safety of RBCs with high concentration of heavy metals to some at-risk groups such as very low weight birth and premature infants have been raised [91].

Smoking, in general, including e-cigarette, vaping, patches and gums of nicotine, is not a criterion for donor screening and deferral. In North America, cannabis smokers are eligible to donate as long as they are not intoxicated, and they can provide informed consent for the donation.

##### Alcohol

Different jurisdictions have different policies for alcohol use before donation. Some accept donors as long as they are not intoxicated and can provide consent, while some centres require 12 or 24 h of abstinence.

Chronic use of alcohol causes increased hemolysis and propensity to osmotic fragility of RBCs [92,93]. Bulle et al. showed that alcoholism caused increased membrane lipid and protein peroxidation and cholesterol/phospholipid ratio. Multiple changes in membrane proteins were also reported, such as increased density of band 3, protein 4.2, 4.9, actin and glycophorins, and deceased in ankyrin [93]. As alcoholism persists, production of normal RBCs impairs and macrocytosis occurs [92].

Contrary to the chronic effects of alcohol, addition of alcohol into RBCs and an immediate evaluation of them showed an improvement in erythrocyte deformability and a decrease in erythrocyte aggregation [94]. This is consistent with the accepted notion that cardiovascular performance benefits from the occasional use of alcohol, with bearing in mind that wine, in particular, is rich in antioxidants, too. Studies that investigated the effect of alcohol on RBC in vitro are very different in design, concentration of alcohol, and exposure method, nonetheless, they suggest improved rheology of RBC after exposure to ethanol [94].

##### BMI/Lipemia

Higher BMI was associated with increased storage hemolysis levels, both in male and female donors in the study by Sparrow et al. on 1734 RBCs [78]. In a study of 18 donors, BMI was found to be positively associated with storage and osmotic hemolysis, and be a significant modifier of storage, oxidative and osmotic hemolysis, explaining 0.2, 4.2%, and 4.5% of the variance, respectively [95]. In a mouse model, a BMI ≥ 30 kg/m^2^ was also associated with lower PTR [95].

The RBCs from donors with BMI of 44 showed dysregulation of antioxidant pathways and nitric oxide metabolism as well as an altered membrane lipid composition, compared to the RBCs from donors with an average BMI of 20 [95]. The above mentioned REDS-III retrospective cohort study investigated any effect of BMI on in-hospital mortality, and they found no association between them [81].

Some blood donors choose to have a full meal or a high-calorie snack before blood donation to prevent feeling light-headed, faint, or nauseous during donation. A high fat meal can transiently increase the plasma lipid levels [96]. In an investigation on obesity-related atherosclerosis, Unruh et al. showed that a high fat diet increased the generation of ROS in murine RBCs [97]. A single high fat meal was also reported to induce nitration of band-3, pathological RBC remodeling, increased intracellular ROS, and oxidative damage to RBC membrane in human [98]. Bashir et al. have shown that resuspension of RBC in lipemic plasma induces hemolysis. Triglyceride-rich chylomicrons were suspected to play a significant role [99]. An earlier study had also demonstrated that exposure to lipemic serum increases the osmotic fragility of RBCs [100].

There is no upper BMI limit for blood donation eligibility as long as the donor is generally healthy and can be safely phlebotomized. There is a minimum weight limit of 50 kg (in UK, Canada and US; different among jurisdictions) for blood donation eligibility. Lipemia is not a criterion for screening of RBCs; however, turbid blood donations are not used for platelet or plasma production.

### 2.3. Medications/Medical Conditions Affecting RBC Quality

In general, every jurisdiction provides a list of medications that prohibit individuals from donating blood (mostly temporarily) and/or a list of commonly used medications that are acceptable when donating blood. Medications, like any other xenobiotics, might change energy metabolism in RBCs, impact their quality and transfusion outcome. In a recent study of REDS-III, for the first time, a high-throughput screening of 1366 FDA-approved drugs showed that around 65% of the tested drugs impacted RBC metabolism [101]. This study investigated RBCs from 250 health donors, and strikingly traces of most common prescription or over-the-counter drugs in the US were detected in at least one donor, which emphasizes the relevance of such investigations [101].

An example of medications that affect RBC quality and are relatively common is exogenous sex hormones. It is estimated that around 16% of American women aged 15–44 use oral contraceptives. Some older women also take estrogens or estrogens in combination with progestins for menopausal hormone therapy. In a study of 6636 female donors, 18–31% (in different races) of premenopausal and 4–8% of postmenopausal females took sex hormones [102]. Hormone intake reduced storage hemolysis in all women and osmotic hemolysis in postmenopausal women. Oxidative hemolysis was increased in premenopausal women who took hormones. This study also showed that progesterone inhibited the Ca^2+^ influx into RBC possibly in an interaction with TRPC6 channels, which could be an explanation for the lower storage hemolysis. Testosterone and 17β-estradiol did not have such effect [102].

There is an increasing trend in receiving testosterone replacement therapies and supplements by young males, and they tend to donate more often than the general population due to erythrocytosis; whether this trend would affect the RBCs quality donated by this group of donors must be investigated [103].

Donor eligibility criteria require donors to feel well at the time of donation. However, affected individuals with some chronic diseases might generally feel well while they are not eligible to donate blood, for example, in case of multiple sclerosis (MS) in UK and Canada. Jurisdictions encourage donors to disclose their diagnosed medical conditions and check for eligibility.

It is expected that RBCs from donors with inflammatory conditions such as autoimmune and hyperallergic predisposition show increased propensity to storage hemolysis. Diabetes, hyperlipidemia, liver and kidney diseases, metabolic syndrome, and hormone imbalance are among the conditions that impact or potentially impact RBC quality [104]. For example, glycosylated hemoglobin A1c in diabetic patients has an increased oxygen affinity and therefore decreased capacity in oxygen delivery to tissues [105] Some diseases such as chronic kidney disease, rheumatoid arthritis, cancer and some genetic traits as discussed above may cause low hemoglobin levels leading to temporary donor deferral.

### 2.4. Inter-Donation Intervals (Frequency of Donation)

Blood centres have set a minimum blood donation interval for donors to prevent iron deficiency and any other unwanted impact on their health. Frequent blood donation was found to decrease the RBC susceptibility to oxidative hemolysis, while it did not affect storage hemolysis and osmotic hemolysis significantly [103]. Depletion of a donor’s iron supply seems to play a role; decreased plasma ferritin results in a decrease in storage hemolysis, oxidative hemolysis and osmotic hemolysis [103]. Whether iron-deficient erythropoiesis in blood donors has any impact on clinical outcome of RBC transfusion is not known yet, however, in a mouse model, RBCs with iron deficiency had a lower 24-h PTR [106]. More data from the REDS-III program on this subject is expected. Frequent donors form a considerable population of donors and can have low ferritin levels even while their hemoglobin levels meet the blood centres requirements.

Minimum blood donation interval for the whole blood is different across jurisdictions. In Canada, for example, it is 8 weeks for males and 12 weeks for females. In UK, it is 12 weeks for males and 16 weeks for females. In the US, it is 8 weeks for all donors.

The only direct measurement performed on donors prior to whole blood donation in many jurisdictions is a hemoglobin measurement. Minimum hemoglobin concentration for blood donation eligibility is 120 or 125 g/L for females and 130 or 135 g/L for males, depending on the jurisdiction. Other than hemoglobin measurement, there are no point-of-care tests or criteria in place to evaluate the quality of donor RBCs.

During storage, the extent of the RBC storage lesion has been mainly assessed by measurement of the percent hemolysis (for research or quality control purpose, not routinely in blood banks), which is affected by genetics of the donor, production-related factors as well as donor individual/environmental characteristics. Hemolysis tests can be performed via automated methods on a drop of blood and have become one of the quality control markers of blood centres in many countries. In Canada, for example, 1% of produced RBC units are evaluated for hemolysis percentage at expiry. To meet the QC requirements, 95% of tested units must have a hemolysis below 0.8%. US Food and Drug Administration has set 1% hemolysis (with 95% confidence that at least 95% of the population meets or exceeds the specification) as one of the in vitro QC criteria. It must be mentioned that units with hemolysis levels above 0.8% are visibly hemolyzed and most likely will not pass the visual inspection before transfusion. This criterion is in place in blood centres mainly to identify any flaws with the manufacturing process. Repeat high hemolysis levels of the donations from same donor can be attributed to a donor factor and are investigated further for the donor characteristics. Other red cells QC parameters in Canada are volume, hematocrit, total hemoglobin, residual leukocyte count and sterility, none of which shed further light on the efficacy of RBCs following transfusion.

While there is much space for improving the current donor blood testing at the time of donation, during storage, and/or prior to transfusion, one must remember the significant role of the recipient in transfusion outcome [107,108], and the final target of achieving a version of personalized transfusion medicine. Roubinian et al. showed that characteristics of both blood donor and recipients besides collection and processing methods are significant predictors of Hb increment following RBCs transfusion. In their study, male donor, female recipient, Rh-D positive donor or recipient, lower BMI of recipient, lower pretransfusion hemoglobin level of recipient and older age of recipient were all associated with higher hemoglobin increment [108].

The ultimate goal of blood centres is to ensure that every patient in need of transfusion will receive high quality and safe blood products. As discussed, the results from multiple clinical trials have suggested that the clinical outcome of RBC transfusion cannot be simply predicted based on RBC storage age or a few donor characteristics. Similarly, in vitro investigations and the new metabolomics findings show that RBC quality cannot be explained by focusing on one marker or molecule.

Nonetheless, identification of the factors affecting RBC quality is crucial for both development of new processing/storage technologies, materials, additives, and diagnostic devices, as well as strategic management of collected blood in blood centres. Many blood centres should have plan for the increasing donor population with genetic traits that affect the RBC quality. New point-of-care diagnostic devices for screening all blood donors or alternatively, the units that go to at-risk recipients might become a routine for blood centres. Electronic or manual tagging of units to provide more information about them to blood banks would be an essential follow up on that. Some centres might divert some trait donors or their donations to a particular blood product. Similarly, as genetic screening for blood group antigens becomes more widespread, it may become more cost effective to look for other genetic differences among donors.

As the information base continues to build, ‘omics’ technologies will eventually lead to the identification of donor biomarkers that will help to identify donors whose RBCs do not store well or have deficiencies that may affect recipient safety. As patient blood management practices continue to lower the demand for RBCs in most high development index countries, it becomes more practical to heighten the focus on product quality rather than simply on sufficiency of supply. Significant latitude has been allowed by regulatory authorities around the acceptable parameters for blood products, and additional scientific information should permit a more rigorous application of a pharmaceutical model.

As more data on the effect of some environmental factors on RBC quality become available, changes in questionnaire or donors might be necessary. Blood centres can consider assigning different shelf-life for the RBC units that are known to store poorly so they would not need to exclude a group of donors, which might cause inventory shortage, while maintaining quality of their products.

## 3. Platelets

Platelets are anucleate cells or cell fragments approximately 2–3 µm that are originally derived from a nucleated precursor, the megakaryocyte, which is resident in the bone marrow. The number of platelets normally ranges from 150–400 × 10^9^/L in the bloodstream and an individual platelet has a lifespan between 8 to 10 days. Platelets (PLT) contain numerous surface receptors, adhesion molecules and granules which can help to perform their essential role in hemostasis. Although they lack a nucleus, platelets contain multiple organelles including mitochondria, endoplasmic reticulum and Golgi apparatus and are metabolically active cells.

Platelet transfusions are given to patients who are actively bleeding, and also as a prophylactic treatment for patients with low platelet counts (thrombocytopenia) or dysfunctional platelets who are at risk of bleeding. PLT transfusion is also used to treat some genetic disorders affecting PLT receptors that render the platelets unable to support normal hemostasis [109,110,111,112]. It should be noted that donor-related variations and also differences in PLT function can translate into clinically applicable outcomes for platelet transfusion recipients. In general, platelet disorders are less common than the RBC disorders described above, and they are more likely to present with clinical symptoms. So, individuals with significant platelet disorders are unlikely to be blood donors. Thus, our following review is not divided by disease as we have done for the red cell section.

Platelet products used for transfusion can either be isolated from whole blood donations using the platelet-rich plasma or pooled buffy coat manufacturing methods or collected as a free-standing platelet product using apheresis machines. Notably, pooled whole blood derived platelet concentrates are normally made from four to six donors unlike apheresis platelets which are a single transfusion dose obtained from one donor. Currently, platelet concentrates (PCs) are stored at 20–24 °C in oxygen-permeable plastic bags with continuous agitation for a maximum of 7 days due to a gradual loss of quality and the potential for bacterial growth. Development of morphologic and metabolic changes during the storage period is known as the platelet storage lesion (PSL). The PSL is reflected in increased PLT activation, PLT granule release and with a decline in PLT responsiveness and metabolic exhaustion.

Quality control (QC) of PCs is essential to ensure suitable benefit of PLT transfusion. Usually, QC tests are conducted on 1% of platelet products at expiry measuring PLT count per unit, pH, residual white blood cells in leukoreduced products and bacterial contamination [113,114]. The quality of PCs is affected by several factors such as preparation methods and storage conditions; however, donor factors that can result in biologic variability and contribute to in vitro platelet quality are also important. Since the production of single donor PCs by apheresis has increased at many blood services worldwide, the donor characteristics are now of greater importance because unlike PCs from pooled donations, any functional defects will not be concealed or diluted within a normal pool of platelets from different donors. Normally pH is checked by QC at the expiry date. Reduced pH is a result of increased lactate production, which is a metabolite produced during the glycolytic pathway to produce more ATP for platelets. Metabolism could differ between various donors and as a result some donors demonstrate steady low pH at an expiry date compared to others. Recently, there are increasing numbers of studies that report donor biologic variation effects on in vitro PCs quality and storage. The volume of clinical data exploring the impact of donor variation on transfusion outcome is much smaller.

### 3.1. Platelets and Hemostasis

Basically, the most significant function of platelets is to initiate hemostasis by swiftly binding to injured blood vessels to stop bleeding. Initially, platelets respond to surface changes or small molecules through outside-in signaling which may lead to full platelet activation, subsequent inside-out signaling and full receptor activation, cytoskeletal rearrangement and granule release. This response is normally initiated by the adhesion of circulating platelets to the exposed collagen within subendothelial extracellular matrix due to vessel injury. Revealed collagen can bind to the platelets indirectly through interaction of GPIb-IX-V surface receptors on platelets with Von Willebrand factor or directly via binding to GP Ia/IIa and GP VI receptors on platelets. Following this initial firm adhesion of platelets to the damaged blood vessel, biochemical processes including signal transduction within platelets induces shape change from discoid to elongated with pseudopod formation associated with the simultaneous release of the contents of their granules, which contain small molecules that can recruit and activate other platelets. Following the formation of a primary clot, the activated platelets start to mediate platelet aggregation via platelet-platelet interactions by incorporating new platelets from circulation through the platelet surface receptor GP IIb/IIIa, which binds fibrinogen [110,111,112]. (See platelet receptor section below for more details).

#### 3.1.1. Metabolomics and Proteomics Data

Investigation of historical QC data of PCs at outdate revealed that different glycolysis rates can result in relatively good or poor storage attributes and PCs can be divided into good (pH_37°C_ > 7) and poor (pH_37°C_ < 6.7) quality groups based the pH on the day after expiry (Day 8) when they are normally tested [115]. Comparison of metabolic characteristics of these PCs demonstrated that the main differences between these two groups is more than 50% higher glucose consumption and lactate production in poor PCs, which indicates a great discrepancy in PLT metabolism and results in higher demand for ATP in this group. It is likely that mitochondrial function plays an important role for poor PLT through increased rates of the citric acid cycle and oxidative phosphorylation. Donors with a history of poor storing PCs compared to good storing PCs, have been suggested to have metabolic perturbation including metabolic syndrome and/or type 2 diabetes, but this hypothesis is lacking adequate data to be supported. These good and poor storing PCs disclose large metabolic differences but relatively small functional differences [115].

Storage is associated with a chain of alterations to the PLT metabolism and proteome. There is a slowly growing literature reporting on the impact of donor sex or age on PLT metabolism and biology in vitro [116,117,118,119]. By investigating PLTs from old donors, these studies suggest that mitochondrial content, metabolism and mitochondrial responses, which are dependent on pro-inflammatory and activating stimuli in PLTs increase with age. Some research has revealed that PLT counts and function seem to be different among sexes with females demonstrating significantly higher age-adjusted mean platelet counts compared to males. Sex dependent differences occur in platelet signal transduction pathways as well [120]. The results of a metabolomics investigation of fresh PLTs from female and male donors revealed a remarkable impact of donor’s sex on PLT metabolism [116]. This study also showed that donors under and over 35 years old demonstrate significantly different metabolomic phenotypes with PLTs from older donors being more metabolically active. The data provide evidence that PLTs from older male donors show increased Krebs cycle metabolism, which is similar to changes seen in murine platelet metabolomic profiles [118]. However; considerably different metabolic phenotypes appear as a result of PLT storage [119]. Stored PLTs metabolism is minimally affected by donor sex in comparison to storage time and storage mostly influences lactate and Krebs cycle metabolites, which are all lower in female donors. Additionally, PLTs from older donors demonstrate significantly higher levels of metabolites. Storage temperature may also be a factor. In comparison to room temperature-stored PLTs, Krebs cycle and pentose phosphate pathway activity in PLTs from old male donors demonstrate consistency with metabolic tendency of cold-stored PLTs.

A useful approach to investigate the presence of different proteins in the PLTs supernatant is proteomics. By using a platelet pool of 22 donors, proteomic analysis of 2501 accurately quantified proteins present in stored PLTs; 21 proteins significantly changed up to 16 days of storage [121]. The level of 18 proteins out of 21 decreased, most of which reside in the α-granules. These data demonstrated limited changes in levels of individual proteins. It has long been postulated that donor sex differences play an important role in proteome composition of plateletpheresis supernatants, especially from female, contributing to adverse transfusion reactions in platelet transfusion recipients. The presence of anti-HLA or anti-neutrophil antibodies in plasma from parous women can result in transfusion-related acute lung injury (TRALI). Furthermore, potential pathways that are involved in the formation of the PSL could affect PLT recipients through febrile non-hemolytic reactions. Proteomic analyses have supported these sex differences [122,123]. These proteomic studies demonstrate an increase of pro-inflammatory factors and activation markers in the plateletpheresis supernatants from female donors over the course of storage. Taken together, these results from stored PLT supernatants illustrate sex-specific traits, which show a sustained increment in levels of several proteins that can contribute to platelet activation, rebuilding of the extracellular matrix and formation of focal adhesions. The interplay between a donor’s sex and subsequent platelet storage can also affect proteins that play a role in coagulation cascades; male PLTs supernatant contains higher levels of coagulation factors, while supernatant from female donors show higher levels of α^2^-macroglobulin.

In addition to proteomic and metabolic studies that explore donor variability, high throughput analysis of genomic information may ultimately prove to be a powerful technique to assess inter donor variability. A recent analysis of *cis*-expression quantitative trait loci (eQTL) examining platelets from 290 normal subjects found 1830 eQTL associated with platelets [124]. As genomic analysis becomes a larger part of transfusion medicine, these technologies can be brought to bear on issues of donor variability for platelet donors [125].

#### 3.1.2. Platelet Receptors

The importance of PLT receptors was briefly discussed in the PLT and hemostasis section above. Here, we will explore more details about those PLT receptors that play an important role in PLT aggregation. Glycoprotein VI (GP VI) on PLT surface functions as the collagen receptor. It recognizes a triple-helical collagen peptide with the recognition motif of glycine-proline-hydroxyproline. A chemically crosslinked collagen-related peptide (CRP) on collagen molecules mediates GP IV adhesion and activates PLTs specifically via GP VI [126]. PLTs of some individuals show reduced aggregation responses which have been attributed to the presence of various GP VI alleles [127]. The GP IV gene is described by high and low frequency alleles named “a” and “b”, respectively. These allelic differences in GP VI appear to affect PLT function and it has been demonstrated that there is a significant difference in the PLT function between “a” and “b” homozygous donors after CRP-XL stimulation, which shows decreased PLT functional responses and expression in donors with low-frequency GP IV allele [127]. Of note, the arrangement of GPO triplets is also important. The minimum effective identifying motifs for GP IV consist of two GPO that could be contiguous or separated by four intervening triplets [126]. Data reflect that the PLT response to cross-linked 2-GPO peptide varied among donors. A remarkable increase in binding was recognized between 2-GPO and 4-GPO content while increasing GPO to 6 and 10 triplets produced a further progressive increment in binding. Thrombus formation analysis by microfluidic assays described subject-dependent differences in thrombus formation or thrombus signature and GPVI-induced platelet activation on collagen surfaces [128]. PLTs of subjects who carry minor alleles of single nucleotide genetic variants in GP VI (rs1613662) and of the megakaryocyte super enhancer variant (rs3557), demonstrate lower levels of PLT GP VI and a concomitant reduced response to CRP-XL and PLT activation. Mean PLT volume and all PLT activation markers correlate positively with the expression levels of surface glycoproteins, which identified them as subject-dependent components [129].

Adenosine diphosphate (ADP) is one of the pivotal substances released from dense granules of PLTs during activation. ADP has an essential function in stimulating PLT aggregation and shape change. ADP signal transduction is regulated by two ADP G-protein-coupled receptors P2Y_1_ (G_q_-coupled) which mediates mobilization of Ca^2+^ and P2Y_12_ (G_i_-coupled), which inhibits the formation of adenylate cyclase. PLT activation is initiated by ADP via P2Y_1_ receptor through shape changes in PLTs and is amplified via P2Y_12_ receptor in a synergic manner, which leads to GP IIb/IIIa activation through phosphoinositide 3-kinase (PI3-kinase) pathway, and PLT aggregation subsequently occurring. There is evidence that any polymorphism of these receptors gene may affect PLT response to ADP [130]. Data illustrate that the P2Y_1_ 1622A > G polymorphism is associated with increased PLT response to ADP in humans; similarly, it has been proved that overexpression of the P2Y_1_ receptor lead to PLT hyper-reactivity to ADP in a transgenic mouse model. The P2Y_12_ receptor gene is specified by 2 haplotypes H1 and H2. Although H2 is a minor haplotype, it is considered to have an increased maximal PLT aggregation ability measured by 4-channel aggregometry and greater inhibition of cAMP accumulation in response to ADP [131]. However, screening of the same P2Y_12_ polymorphisms in other investigation demonstrated no effect on PLT reactivity by flow cytometry due to fibrinogen binding to GP IIb/IIIa. By using flow cytometry method for evaluation of PLT response to ADP via fibrinogen binding to activated GP IIb/IIIa, it has been unraveled that there is a positive correlation between PLT response and increasing age as well as density of GP IIb/IIIa receptors on PLTs, whereas current smokers demonstrated negative relation [130,131,132].

Since PLT aggregation is mediated by fibrinogen binding to the GPIIb/IIIa receptor of platelets, heritability of PLT aggregation responses to epinephrine, ADP and collagen lag time was studied along with fibrinogen receptor genotype based on sibling correlations [133]. Increased PLT aggregability has been shown due to the presence of PI^A2^ allele of GP IIIa by gradual lower concentrations for epinephrine and ADP [134]. However, further studies represented that the PI^A2^ allele of GP IIIa and the fibrinogen Hind III β-148 genotype play a small role in PLT aggregation, which shows that more investigation is needed for other genetic variants of proteins involved in the PLT adhesion or aggregation process. It should also be noticed that in order to produce irreversible PLT aggregation, there is a significant interindividual variations in the concentration of required ADP. It was demonstrated that increasing age is associated with decreased ability of PLT to aggregate to epinephrine and ADP, while increased triglyceride and decreased HDL levels are significantly related with lower PLT aggregation to epinephrine and collagen lag time [133,134].

#### 3.1.3. Platelet Function

Researchers are trying to figure out how PLT function changes, owing to various individual characteristics [135,136,137,138]. One of the most important factors is the genetic variability of the PLT donors. There is a broad inherent variation in the PLT response among donors, which shows that this process is genetically controlled. Individuals can be categorized as hypo, intermediate, or hyper responders to ADP and collagen agonists [135,137]. Donors with more responsive PLTs before donation, show more activated PLTs harvested from plateletpheresis units after donation compared to both low and intermediate responders [138]. Of note, the PLT function as a potential influence on donated PLT’s quality stays stable over time, with up to 68 months of regular donations [136]. Similarly, the outdate pH of apheresis platelets is very consistent for an individual donor, and donors whose donations fail pH on one occasion are likely to have this characteristic repeatedly [139].

By using the PFA-100 analyzer, which is an instrument that provides repeatable measurements of PLT function under a high-shear system through closure time (CT) evaluation induced by collagen-epinephrine and collagen-ADP in consecutive donations, it has been shown that there is an interindividual variability of CT values for collagen-epinephrine stimulated PLTs in healthy PLT donors which is similar to aspirin-like defect detection and it is often transient [140]. By measurement of serum thromboxane B2 levels, it is believed that this increased CT value among these donors is comparable with cryptic intake of aspirin and nonsteroidal anti-inflammatory drugs, which could be included in over-the-counter drugs that donors may unknowingly ingest [141]. It has been demonstrated that sex or age does not affect the CT values obtained from both collagen-epinephrine and collagen-ADP cartridges [142]. Furthermore, the CT-value is modulated by blood groups and individuals with the O blood group show slightly abnormal CT values due to a lower amount of VWF antigen levels in their blood. According to older data evaluated by aggregometry, female PLTs were more responsive and in both sex groups PLT responsiveness increases with age [143].

Stored normal PLTs can regenerate their function to some extent when transfused. However; defective PLTs from donors seem to remain dysfunctional [144]. Although it has been established that variation in PLT responses is in part inherited, some factors related to blood donors such as diet, drugs, smoking and exercise can worsen the PLT’s acquired defects. However, typically in platelet collection for transfusion products, PLT function is not assessed before donation and donors are just asked about any family history of bleeding and recent consumption of any medications. This approach may eliminate donors with severe PLT defects but not donors with mild or transient defects.

Although it has been hypothesized by some researchers that thromboelastography (TEG) assays on plateletpheresis donors might be helpful to evaluate donor-specific and storage-induced responses, suggesting that clot retraction could be a qualitative TEG variable of donor-related, this approach needs standardization to be able to discriminate donor-specific differences [145].

### 3.2. Transfusion Outcome

Platelet function varies among normal individuals; this variation is stable over time within the same individual. There is relatively little literature that addresses whether donor-related variation in PLT function affects patient outcome following transfusion. The ability to predict the in vivo PLT recovery, survival and function using in vitro PLT function measurement is still controversial. There are some reports suggesting that the increased activation that occurs during storage of PLTs correlates negatively with post-transfusion survival, while other findings imply this is not the case [146]. According to evidence suggesting that phenotypic and proteomic changes of PLTs during storage could be in part determined by the donor’s genotype, it is important to understand that variation occurs not only in donors, but also in recipients. Overall, the data support the concept that donor-related variations and also differences in PLT function can translate into clinically applicable outcomes for platelet transfusion recipients.

Platelet count increment, usually reported as a Corrected Count Increment (CCI), is used as the endpoint to answer the question whether differences in the level of PLT responsiveness of donors to agonists in vitro can affect clinical outcome. A lower CCI following transfusion may mean that PLTs from high responder donors are cleared more rapidly from circulation. In one study of platelet transfusion outcomes in hematology-oncology patients, in vitro agonist responsiveness was not associated with transfusion outcome, suggesting that this may not apply to prophylactic transfusion [146]. In addition, the bleeding prevention ability of PLTs did not differ between these two groups [147].

PLT units with decreased pH levels less than 6.2 at room temperature are demonstrated to have poor survival post-transfusion and most likely supply little hemostatic efficacy while using radiolabeled recovery and survival of autologous PLTs for measurement of in vivo PLT recovery. However, there is no relationship between post-transfusion viability when in vitro pH22 °C is at least 6.2 [148].

PLT morphology, extent of shape change and the expression of surface markers such as CD42b and P-selectin, correlate relatively weakly with post-transfusion viability, which is variable from one donor to another. Investigation of autologous infusion of radiolabeled PLTs in order to study post-transfusion viability and metabolomic analysis of the stored PLTs revealed multiple discrete metabolites that play a role for post-transfusion PLT recovery or survival [149]. Purine deamination has been confirmed as a marker of post-transfusion recovery [116]. Metabolic pathways such as pentose phosphate and markers of medium chain fatty acyl-carnitines and long chain fatty acid catabolism are correlated positively to post-transfusion recovery [149]. The glutathione homeostasis pathway, redox metabolism and glutaminolysis are significantly positively related to long term survival in circulation upon transfusion. Similar to red blood cells, glucose 6-phosphate dehydrogenase (G6PD) deficiency negatively affects storability and post-transfusion performance of PLTs. Metabolism markers of Krebs cycle, oxidant stress, short chain-fatty acyl-carnitines and arginine/tryptophan are also negatively related to post-transfusion recovery, while the level of Krebs cycle metabolites and lactate are negatively correlated to long term survival of PLTs in circulation after transfusion [149].

Investigation of potential platelet protein biomarkers by proteomics analysis demonstrated that regulatory proteins that are relevant to the function of transfused PLTs are associated with mediating in vivo transfusion outcome. Higher expression of JAM-1 and tropomyosin-4 (TPM-4) was confirmed by immunoblot analyses for samples from donors with worse transfusion outcomes in recipients [150]. Protein levels of Rap1 and RhoGDI during storage demonstrated either no increase or decreased levels and correlated with impaired PLT recovery and survival [151].

Although no significant differences as a function of sex or age is seen in post-transfusion recoveries, by investigating PLTs at the end of the allowable storage period, post transfusion survival of these cells tend towards reduction in older male donors. Interestingly, ribose phosphate and 6-phosphogluconate, metabolites in the pentose phosphate pathway are considered to have a positive correlation to age and post-transfusion recovery [116].

Some factors such as smoking, which can affect stored PLTs by influencing the redox homeostasis and mitochondrial metabolism, may result in negative effect on post-transfusion recoveries and survival. However, the PLT levels of cotinine as a nicotine metabolite have not been observed to demonstrate remarkable correlation with post-transfusion recoveries and survival [116,149].

As can be seen from the review above, there is a lack of consistency among reports in the literature about the specific platelet characteristics that affect transfusion outcomes. Almost all of the clinical studies have been conducted in stable hematology-oncology patients receiving prophylactic platelet transfusions. Since a significant proportion of platelet transfusions are given to actively bleeding patients, future research should focus on more diverse categories of platelet transfusion recipients.

## 4. Conclusions

Appreciation of the role of donor variability in the determination of blood product quality continues to attract research interest. It is now clear that all blood products are not created equal and that the quality of blood components, and the outcome of transfusions, is dependent on factors related to the donors. These may be inherent genetic characteristics or the influence of non-genetic factors including age and lifestyle choices. With an increasing accumulation of data used to clarify which markers matter the most, we will be able to establish a biomarker profile to assist in understanding whether the products from some donors will require reduced storage duration or other shifts made to accommodate these donor variations and their impact on blood product quality. As this field evolves, we will reach a time when these donor characteristics are used to manage donations to the optimum benefit of transfused patients.

## Figures and Tables

**Table 1:** Some of the similarities and differences between G6PD-deficient and G6PD-normal RBCs during blood bank storage [31,32,33].

Some Findings from the Storage of G6PD-Deficient and G6PD-Normal RBCs	
Similarities	Differences
Storage hemolysis ATP (throughout storage) Lactate Glucose pH Na^+^ Morphology Microvesiculation ROS accumulation Ca^2+^, up to d42 No activity of oxidative arm of PPP after 6 weeks of cold storage	**Fresh (before cold storage) G6PD-deficient RBCs had** No activity of oxidative arm of PPP while G6PD-normal RBCs did Higher ATP levels; Defect in G6PD ⇨ ↑available G6P ⇨ ↑glycolysis ⇨ ↑NADH ⇨ ↑ATP; Less conversion of pyruvate to lactate (a NADH-consuming reaction) ⇨ ↑ATP **During storage, G6PD-deficient RBCs had** Lower levels of methemoglobin ↑NADH ⇨ ↑cytochrome-b5 reductase activity ⇨ ↑methemoglobin reducing reaction Lower levels of GSH, hexose sugar alcohol, and dihydroxynonene; ↓NADPH ⇨ ↓NADPH-dependent reactions that generate these molecules Lower methionine, S-adenosyl-L-methionine, methylenetetrahydrofolate; Altered one-carbon and sulfur metabolism Lower levels of urate, glutamine, glutathionylcysteine; Altered glutathione homeostasis and antioxidant defenses Increased pyruvate level; Alteration in glycolysis ⇨ ↑pyruvate A side product of compensatory mechanism for generation of NADPH by conversion of malate to pyruvate Increased Biliverdin ↓NADPH ⇨ ↓NADPH-dependent conversion of biliverdin to bilirubin Lower levels of free fatty acids (except for oleate and linoleate) and their oxidized derivatives (e.g., HPETE/LTB4 or isobaric isomers)

## Data Availability

All data described in this review can be found in the papers in the reference list.
